# Simultaneous integrated boost therapy of carcinoma of the hypopharynx/larynx with and without flattening filter - a treatment planning and dosimetry study

**DOI:** 10.1186/s13014-017-0850-8

**Published:** 2017-07-05

**Authors:** Barbara Dobler, Tina Obermeier, Matthias G. Hautmann, Amine Khemissi, Oliver Koelbl

**Affiliations:** Department of Radiotherapy, Regensburg University Medical Center, Regensburg, Germany

**Keywords:** Hypopharynx carcinoma, Larynx carcinoma, Simultaneous integrated boost, Flattening filter free, Peripheral dose, Radiation induced second cancer

## Abstract

**Background:**

The aim of this study was to investigate if the flattening filter free (FFF) irradiation mode of a linear accelerator (linac) is advantageous as compared to the flat beam (FF) irradiation mode in intensity modulated radiation therapy (IMRT) and volumetric modulated arc therapy (VMAT) for carcinoma of the hypopharynx / larynx.

**Methods:**

Four treatment plans were created for each of 10 patients for an Elekta Synergy linac with Agility collimating device, a dual arc VMAT and a nine field step and shoot IMRT each with and without flattening filter. Plan quality was compared considering target coverage and dose to the organs at risk. All plans were verified by a 2D–ionization-chamber-array and delivery times were compared. Peripheral point doses were determined as a measure of second cancer risk. The Wilcoxon test was used for statistical analysis with a significance level of 0.05.

**Results:**

Plan quality was similar for all four treatment plans without statistically significant differences of clinical relevance. The clinical goals were met in all plans for the PTV-SIB (V_95%_ > 95%), the spinal cord (D_1ccm_ < 45 Gy) and the brain stem (D_1ccm_ < 48 Gy). For the parotids, the goal of D_50%_ < 30 Gy was met in 70% and 60% of the plans for the left and right parotid respectively, and the V_95%_ of the SIB reached an average of 94%. Delivery times were similar for FF and FFF and significantly decreased by around 70% for VMAT as compared to IMRT. Peripheral doses were significantly reduced by 18% in FFF mode as compared to FF and by 26% for VMAT as compared to IMRT. Lowest peripheral doses were found for VMAT FFF, followed by VMAT FF.

**Conclusions:**

The FFF mode of a linear accelerator is advantageous for the treatment of hypopharynx/larynx carcinoma only with respect to reduction of second cancer induction in peripheral organs for the combination of Elekta Synergy linacs and Oncentra® External Beam v4.5 treatment planning system. This might be of interest in a therapy with curative intent.

## Background

Overall survival of patients with hypopharyngeal and laryngeal carcinoma improved over the last years due to new cytostatic substances [1, 2] and improved radiation therapy techniques [3]. Intensity modulated radiotherapy (IMRT) and volumetric modulated therapy (VMAT) have emerged as standard external beam radiotherapy techniques to treat head and neck carcinoma due to significant benefits in quality of life and survival as compared to conventional conformal radiation therapy [3]. An increase in loco-regional control may be achieved using dose escalated fractionation schemes [3, 4]. Simultaneous integrated boost techniques are shortening overall treatment times and have shown to be a feasible and save alternative to sequential boost irradiation [5–10].

During the last years, standard linear accelerators with large field sizes have been installed with an opportunity to irradiate patients without a flattening filter in the beam path to increase dose rate and reduce beam-on time [11]. Since the flattening filter is a source of scatter radiation, its removal has the positive side effect of lowering out-of-field doses [12]. A variety of planning studies has shown that the flattening filter free (FFF) mode of a linac allows reducing delivery times in stereotactic treatments with high fraction doses [13–21]. The challenge in FFF treatment planning is, however, planning for large targets due to dose decrease with distance from the central beam axis. In addition delivery times are influenced to a larger extent by mechanical constraints of the gantry and the multi leaf collimator (MLC) in normo-fractionated treatments with fraction doses around 2 Gy. First planning studies for larger target types showed advantages for FFF for some combinations of target type, treatment technique, planning system and linac, whereas no advantage or even disadvantages could be observed in others [22–28], confirming the need of further investigations depending on target localization and equipment used, as already pointed out in [12, 29].

The purpose of this study was therefore to evaluate if the FFF irradiation mode is advantageous with respect to plan quality, delivery time and peripheral dose as a measure of excess second cancer risk as compared to the flat beam (FF) irradiation mode in IMRT and VMAT for carcinoma of the hypopharynx/larynx for the combination of Elekta Synergy linacs and Oncentra External Beam v4.5 treatment planning system.

## Methods

### Patients and dose prescription

CT data of 10 patients with carcinoma of the hypopharynx/larynx were selected from our treatment database and included in this study. All patients had locally advanced tumors of Union Internationale Contre le Cancer (UICC) stage IVa or IVb. A CT scan was used for delineation of target volumes including information of MRI and/or PET-CT scans. All patients received bilateral cervical radiotherapy. Two planning target volumes were defined as follows: The clinical target volume CTV 1 includes the primary tumor (gross tumor volume (GTV)) with a 1 cm margin or with anatomically corrected margins adjacent to uninvolved areas and including the involved lymph nodes as well as the levels with high risk of involvement. The clinical target volume CTV 2 contains the CTV 1 and the lymph node levels with moderate risk of involvement. The planning target volumes are derived from the clinical target volumes adding a margin for systematic and random setup errors and internal motion of 4 mm and are named SIB (simultaneous integrated boost volume derived from CTV 1) and PTV (planning target volume derived from CTV 2) respectively. The skin was excluded from the PTV and SIB volumes. The spinal cord, brainstem, parotid glands, and oral cavity were defined as organs at risk (OAR) [7, 30].

Dose prescription for the simultaneous integrated boost radiation therapy was an average dose of 66 Gy in 30 fractions of 2.2 Gy to the SIB and 54 Gy in 30 fractions of 1.8 Gy to the PTV-SIB according to [6, 30–32]. Target coverage, represented by the volume of the SIB and PTV covered by 95% of the respective prescription dose (V_95%_) is generally aimed at 95%. Due to the proximity of the target volumes to the patient outline, i.e. the body contour, however, a V_95%_ of 90% is considered acceptable in this case to avoid severe radiodermatitis. For the organs at risk dose volume tolerances listed in Table 1 in column “Goal” were chosen as clinical goals. For the parotid salivary glands, the mean dose should be kept as low as possible to maintain salivary function [33]. In the literature a goal of D_50%_ < 30 Gy at least in one gland if possible depending on the overlap of parotid glands and PTV has been used for optimization and evaluation [7, 32, 34]. The dose to the oral cavity should be reduced as much as possible to reduce the risk of severe mucositis [7, 32].

**Table 1:** Comparison of plan quality

			IMRT		VMAT	
Parameter		Goal	FF	FFF	FF	FFF
SIB	V_95%_	> 95%	93.7 ± 1.2	93.7 ± 1.3	94.5 ± 1.6	94.2 ± 1.9
	HI		0.15 ± 0.03	0.15 ± 0.03	0.14 ± 0.03	0.15 ± 0.04
	CI	> 0.7	0.83 ± 0.04	0.83 ± 0.04	0.87 ± 0.1	0.83 ± 0.05
PTV - SIB	V_95%_	> 95%	98.8 ± 0.3	**99.0 ± 0.3**	99.0 ± 0.3	98.9 ± 0.6
	HI		0.23 ± 0.02	**0.22 ± 0.02**	0.22 ± 0.03	0.24 ± 0.04
PTV	CI	> 0.7	0.79 ± 0.03	0.78 ± 0.03	0.76 ± 0.03	0.76 ± 0.03
Spinal Cord	D_1ccm_	< 45 Gy	31.7 ± 2.1	31.5 ± 1.7	32.2 ± 1.8	31.7 ± 1.4
Brain Stem	D_1ccm_	< 48 Gy	34.6 ± 4.7	34.6 ± 5.0	**39.5 ± 4.9**	42.1 ± 4.5
Parotid Left	D_50%_	< 30 Gy	29.0 ± 4.8	28.3 ± 5.2	29.2 ± 5.6	29.9 ± 6.2
Parotid Right	D_50%_	< 30 Gy	30.5 ± 4.8	29.5 ± 4.4	29.7 ± 4.2	29.6 ± 4.3
Oral Cavity	V_60%_		50.8 ± 15.6	51.8 ± 16.0	52.0 ± 15.2	54.0 ± 17.0

Mean values and standard deviation of the dose volume parameters for FF and FFF mode averaged over all patients separated by the treatment technique. Dose values are given in Gy, volumes in % of the structure volume. HI stands for homogeneity index, CI for conformity index. Bold values indicate statistically significant superior values in the comparison of FF vs FFF

### Linear accelerator and treatment planning system

Treatment planning is performed with Oncentra External Beam v4.5 (Nucletron, an Elekta Company) for a Synergy linear accelerator with Agility collimating device (Elekta AB, Stockholm, Sweden) and 6 MV photons with flattening filter or without. The FFF beams were energy-matched to the FF beams with respect to percentage depth dose and quality index as described in [29, 35]. The multi leaf collimator consists of 80 leaf pairs of 5 mm width at isocenter. The maximum nominal dose rate is 500 monitor units (MU) per minute (min) in FF Mode and 1700 MU/min in FFF mode.

### Treatment planning

For each patient four treatment plans were created, using the two treatment techniques IMRT and VMAT each in the two irradiation modes with and without flattening filter, in the following referred to as IMRT FF, IMRT FFF, VMAT FF, and VMAT FFF. The IMRT plans consist of nine equispaced beams, minimal segment size was 9 cm^2^ and maximal number of segments allowed was 90. The relatively high number of segments was allowed due to the complexity of the simultaneous boost irradiation which requires a steep dose gradient between the PTV and SIB. This number is used as upper limit and is not exploited by the optimizer implemented in Oncentra, if similar plan quality can be achieved with a lower number of segments. Minimal number of MU per segment is 4 due to the determined stability of the beam for 4 MU and higher. The VMAT plans consist of two full rotations, since it was not possible to achieve the required plan quality with a single arc. Gantry spacing between two control points was 4°, collimator angles ranged from 0° to 45° for both techniques. The isocenter was located centrally in the PTV for all plans. Suitable dose volume objectives (DVO) and weights were determined creating plans in FF mode which met the goals and then transferred to the FFF plans. Identical DVO and weights were used for optimization of all plans. All plans were accepted for treatment by a specialized radiation oncologist and normalized to the average dose in the SIB.

### Dosimetry and delivery

All 40 plans were dosimetrically verified using the MatriXX Evolution™ 2D–ionization-chamber-array (IBA Dosimetry, Schwarzenbruck, Germany) set up in between slabs of a RW3 phantom (PTW, Freiburg, Germany). All beams were irradiated and evaluated as complete plan verification. In addition a point dose measurement was performed 31 cm cranial of the isocenter using the 0.3 ccm ionization chamber (IC) of type M23332 in combination with the dosimeter Unidos (PTW, Freiburg, Germany) in low dose rate mode (range 1.3 mGy/min – 1 Gy/min) to assess peripheral dose as a measure of excess second cancer risk. The energy dependence is within ±2% in the kV range of the scatter radiation according to the manual. Measurements of corresponding FF and FFF plans were performed without repositioning of the IC to avoid uncertainties due to inaccurate positioning. The measurement setup has been described in detail in the framework of another study [36]. During phantom measurements delivery times were recorded from first beam on to last beam off.

### Evaluation

Plan quality was assessed by analysis of the dose volume histogram (DVH) for the SIB, the PTV and the organs at risk. Target coverage was represented by the volume of the SIB and PTV covered by 95% of the respective prescription dose (V_95%_). The homogeneity index defined as HI:= (D_1%_ - D_99%_) /D_50%_ was recorded for the SIB and for the PTV excluding the SIB (PTV-SIB), to exclude the high dose region of the SIB from the analysis of the PTV. The conformity index was defined as as CI:= V_95%_^2^/ (TV ⋅ PIV) according to Paddick et al. [37]. Here TV means the volume of the structure, PIV the total volume covered by 95% of the prescription dose. For the spinal cord and the brainstem, the dose to 1 ccm (D_1ccm_) was evaluated as a measure of clinically relevant maximum dose. For the parotids the median dose (D_50%_) was recorded and compared to the clinical goal of <30 Gy [6, 7, 32]. For the oral cavity V_60%_ was analysed. In addition the number of MU required per fraction dose and the number of IMRT segments was compared.

For evaluation of plan verification gamma indices as defined by Low et al. [38] were calculated with a dose tolerance of 3% of the maximum dose and 3 mm distance to agreement. The agreement between dose calculations and measurements is considered acceptable if at least 95% of the pixels with a dose value of ≥10% of the maximum dose have a gamma value ≤1 as recommended by the AAPM TG119 [39, 40].

In the low dose region below 1 Gy the excess absolute risk of developing radiation-induced second cancer (EAR) has been shown to be linear with dose in an extensive analysis of the survivors of the atomic bombs of Hiroshima and Nagasaki [41, 42]. Peripheral IC point dose measurements were compared for the two irradiation modes FF and FFF for the whole treatment of 30 fractions. Since dose values are well below 1 Gy in this region, the ratio of peripheral doses in FFF and FF mode corresponds to the same ratio of EAR in this dose region. The application of correction factors to achieve absolute doses with high accuracy is not required, since the goal is to estimate the ratio of EAR in the two irradiation modes and all correction factors cancel out during division. The uncertainty was estimated to be within ±3%, taking uncertainties due to differences in the energy spectra of the scatter radiation between FF and FFF and statistical uncertainty into account. For statistical analysis the two sided Wilcoxon signed rank test implemented in IBM SPSS® Statistics 23.0 (IBM Corporation) was used to detect relevant differences of the size of a standard deviation or larger (effect size 1) with a significance level of 0.05.

## Results

Since the main subject of the study was the comparison of the two irradiation modes FF and FFF, details about statistical significance are listed in the tables for this comparison. Significant differences between IMRT and VMAT are mentioned in the text but not listed in detail in the tables for the sake of clarity.

### Plan quality

**Fig. 1:**
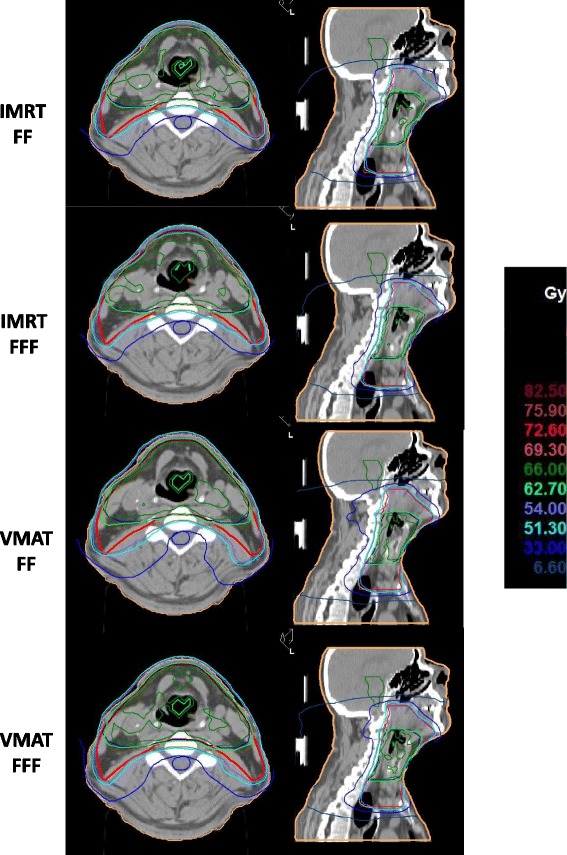
Comparison of dose distributions. Comparison of dose distributions in one transversal (left) and one sagittal (right) slice for a representative case. The PTV is drawn in red, SIB dark blue, left parotid orange, right parotid olive, spinal cord cobalt blue, brainstem green, and patient outline orange. The light blue isodose line represents 95% of the prescription dose to the PTV, the green isodose the 95% of the prescription dose to the SIB

**Fig. 2:**
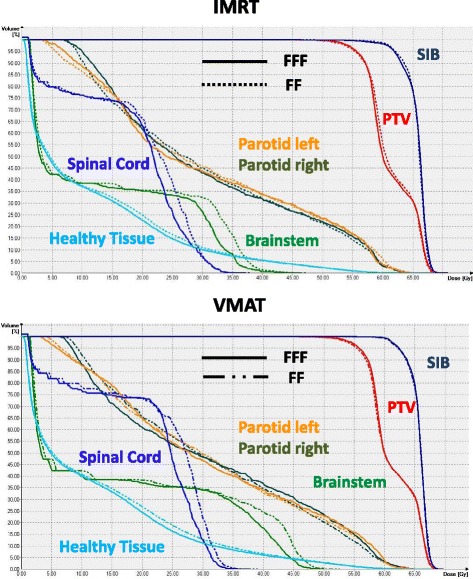
Comparison of dose volume histograms. Comparison of dose volume histograms for the case of Fig. 1. Top IMRT, bottom VMAT. Solid lines represent FFF, dotted lines FF. The PTV is drawn in red, SIB dark blue, left parotid orange, right parotid olive, spinal cord cobalt blue, brainstem green, and healthy tissue (patient outline excluding targets) light blue

Acceptable plan quality was achieved with all four treatment plans. Details about DVH parameters averaged over all patients are given in Table 1. A comparison of dose distributions and dose volume histograms is shown in Figs. 1 and 2 for a typical case. The goals listed in Table 1 were met in all 40 plans for the PTV-SIB, the spinal cord and the brain stem. For the parotids, the goals were met in 70% and 60% of the plans for the left and right parotid respectively, and the V_95%_ of the SIB reached an average of 94% without significant differences between FF and FFF. FFF led to significantly higher target coverage and homogeneity in PTV-SIB when IMRT was used as treatment technique. Differences were, however, small with <1% for V_95%_ and 4% for HI. For VMAT the maximum dose D_1ccm_ to the brain stem was significantly increased by 7% in FFF mode, but kept well below the clinical goal of 48 Gy. All other dose-volume-parameters evaluated in this study were similar without significant differences between FF and FFF. Comparison of the two planning techniques showed significantly higher target coverage and homogeneity for VMAT in FF mode and lower OAR and normal tissue doses for IMRT in both irradiation modes. Differences in target metrics were, however, marginal between all four plans.

### Dosimetry and delivery

All 40 plans passed the gamma evaluation. No significant difference could be observed in the passing rate between FF and FFF or IMRT and VMAT. Peripheral point doses were significantly reduced by 18% in FFF mode for both planning techniques. In comparison of the planning techniques peripheral doses were significantly lower for VMAT in both irradiation modes with an average reduction of 26% in FF mode and 27% in FFF mode as compared to IMRT. The significantly lowest peripheral dose was found for VMAT FFF. Delivery times increased slightly but significantly in FFF mode by 4% in case of IMRT but were similar in both irradiation modes in case of VMAT. For IMRT around 3 fold delivery times were required as compared to VMAT in both irradiation modes. Detailed information about dosimetry and delivery can be found in Table 2.

**Table 2:** Comparison of delivery

Parameter	IMRT FF	IMRT FFF	VMAT FF	VMAT FFF
Passing Rate of Gamma Evaluation (%)	98.5 ± 1.3	97.6 ± 2.0	98.6 ± 0.9	98.9 ± 0.9
Delivery Time (min:s)	**9:27 ± 0:34**	9:49 ± 0:28	2:52 ± 0:07	2:53 ± 0:05
Measured Peripheral Dose 30 frac (mGy)	209 ± 36	**170 ± 34**	155 ± 42	***127 ± 38***
Monitor Units	859 ± 70^a^	1181 ± 120	571 ± 63^a^	694 ± 59
Segments (IMRT)	71 ± 4^a^	79 ± 3	-	-

Mean values and standard deviation of delivery time, monitor units and segments for FF and FFF mode averaged over all patients. Bold values indicate statistically significant superior values in the comparison of FF vs FFF. Bold-italic values indicate best values in the comparison of all planning techniques and irradiation modes. ^a^ indicates statistical significance in the comparison of FF vs FFF without judgement, since the number of monitor units and the number of segments are no measures of quality

## Discussion

The aim of this study was to investigate if the FFF irradiation mode of a linear accelerator is advantageous as compared to FF for IMRT and VMAT of carcinoma of the hypopharynx and larynx. The results of the study show, that it was possible to create acceptable treatment plan quality for IMRT and VMAT without a clear advantage of one of the two irradiation modes: The target goals and OAR tolerances listed in Table 1 were achieved in all cases for the PTV-SIB, the spinal cord and the brain stem. A few significant differences were observed in the PTV-SIB and the brain stem, but the clinical goals were achieved in all cases and the differences are therefore not considered clinically relevant. The goal of D_50%_ < 30 Gy was achieved averaged over all plans, but exceeded in 30% and 40% of the plans for the left and right parotid respectively, without significant differences between FF and FFF. The reason that the goal of D_50%_ < 30 Gy was not achieved in all patients was a large overlap of the parotid volume and the PTV in these patients and the fact, that unilateral sparing of one parotid was in general not applicable due to bilateral lymph node involvement and therefore bilateral extension of the PTV and SIB. The results were in concordance with another dose escalation study, which reported mean and median doses >30 Gy to the parotids for a similar fractionation scheme [6] and were accepted since the goal of parotid sparing must not compromise target coverage as also stated in [33]. Target coverage in the SIB represented by V_95%_ reached an average of 94% in both irradiation modes. This was accepted because of the proximity of the target to the patient outline and because special attention was given to the dose conformity to avoid large high dose volumes outside the targets, which reached excellent values well above the goal of 0.7 in all cases. Peripheral point dose measurements in the region well below 1 Gy showed a significant reduction by 18% in FFF mode. Lowest peripheral doses were measured for VMAT FFF, with a reduction of 40% as compared to IMRT FF. Due to the linearity of the excess absolute risk of developing second cancer with dose in this dose region, this corresponds to a reduction of the EAR of 18% and 40%, respectively. This can be explained by reduced head scatter if the flattening filter is removed.

Delivery times increased slightly but significantly in case of IMRT by 22 s from 9:27 min to 9:49 min corresponding to 4% when FFF mode was used. In case of VMAT delivery times were similar with a mean of 2:52 min for FF and 2:53 min for FFF. The increased delivery time for FFF as compared to FF for IMRT can be explained by the fact that a larger number of segments is required to achieve a homogeneous dose distribution. In step and shoot IMRT the beam is turned off during mechanical movements of the linear accelerator and the influence of the dose rate is small for fraction doses of about 2 Gy as compared to higher fraction doses used in stereotactic treatments. For VMAT, however, the beam stays on during mechanical movements and the increased number of MU observed in this study with a factor of 1.3 might theoretically be overcompensated by the dose rate which is increased by up to a factor of 3.4 in FFF mode. The maximum dose rate is, however, not exploited throughout a VMAT treatment, since the gantry speed is limited to 1 round per minute. A minimum irradiation time of 120 s is therefore required for a dual arc VMAT independent on the maximum dose rate available. For the equipment used in this study, auto-sequences are allowed for IMRT but not for VMAT. Therefore user interaction is required at the end of the first arc for recording of the first and loading of the second arc, which takes additionally around 25 s. Thus the minimal total delivery time required theoretically for a dual arc VMAT is around 145 s. This is nearly achieved in the VMAT FF plans in the study presented here, with an average of 172 s and a minimum of 159 s. Therefore the available higher dose rates in FFF mode are not fully exploited and total delivery times are not further decreased.

A recently published study evaluated FFF for IMRT and mArc treatment of hypopharynx carcinoma with a prescription dose of 50 Gy to the PTV and found no advantages with respect to plan quality but a slight decrease in treatment time [28]. Lowest delivery times were achieved with mArc FFF ranging from 5:25 to 5:37 min for a 2 Gy fraction dose. Irradiation times were substantially lower in our study for VMAT in both irradiation modes despite of higher fraction doses of 2.2 Gy to the SIB. The reason for this can be found in the concept of the mArc technique in which continuous gantry rotation is combined with a step and shoot like separation of irradiation and MLC movement. This concept does not allow equally high efficiency as the fully dynamic VMAT technique used in our study presented here.

The results of our study confirm that the results of planning studies for a certain combination of equipment and tumour site cannot be generalized: A clear advantage for irradiation in FFF mode, as it has previously been found for the same combination of linear accelerator and treatment planning system for the re-irradiation of spinal metastases [36], and for a another treatment planning system and treatment of breast cancer [27] could not be detected in the study presented here: Differences in plan quality and delivery were negligible between the two irradiation modes. The only advantage of FFF mode observed in our study was the reduction in peripheral dose corresponding to a decreased risk of radiation induced second cancers in peripheral organs as it also has been observed for the treatment of breast cancer in a previous study [43]. This, however, is of interest only for a therapy with curative intent, as it is the case e.g. for virus associated tumours.

## Conclusions

The flattening filter free mode of a linear accelerator is advantageous for the treatment of hypopharynx/larynx carcinoma only with respect to reduction of second cancer induction in peripheral organs, which might be of interest in a therapy with curative intent. No advantage could be observed with respect to plan quality or delivery times.

## Abbreviations

CI: Conformity Index
DVH: Dose Volume Histogram
DVO: Dose Volume Objective
D_x_: Dose to volume x of the structure
EAR: Excess Absolute Risk of developing radiation-induced second cancer
FF: Flattening Filter
FFF: Flattening Filter Free
HI: Homogeneity Index
IMRT: Intensity Modulated Radiation Therapy
min: Minutes
PIV: Total volume of the 95% isodose
PTV: Planning Target Volume
s: Seconds
SIB: Simultaneous Integrated Boost volume
TV: Volume of the PTV,
VMAT: Volumetric Modulated Arc Therapy
V_x%_: Volume covered by x% of the prescription dose
